# Accounting for arterial and capillary blood gases for calculation of cerebral blood flow in preterm infants

**DOI:** 10.1007/s00431-022-04392-0

**Published:** 2022-02-12

**Authors:** Silke Brodkorb, Irina Sidorenko, Varvara Turova, Esther Rieger-Fackeldey, Ursula Felderhoff-Müser, Andrey Kovtanyuk, Renée Lampe

**Affiliations:** 1 Department of Neonatology, Munich Clinic Harlaching, Sanatoriumsplatz 2, Munich, 81545 Germany; 2 Chair of Mathematical Modelling, Mathematical Faculty, Technical University of Munich, Boltzmannstr. 3, Garching, 85748 Germany; 3 Research Unit for Pediatric Neuroorthopedics and Cerebral Palsy of the Buhl-Strohmaier Foundation, Orthopedic Department, School of Medicine, Klinikum rechts der Isar, Technical University of Munich, Ismaninger Str. 22, Munich, 81675 Germany; 4 Department of Pediatrics, School of Medicine, Klinikum rechts der Isar, Technical University of Munich, Ismaninger Str. 22, Munich, 81675 Germany; 5 Department of Pediatrics I, Neonatology, Pediatric Intensive Care, Pediatric Neurology, University Hospital Essen, University Duisburg-Essen, Hufelandstraße 55, Essen, 45147 Germany

**Keywords:** Preterm birth, Intraventricular hemorrhage, Cerebral blood flow, Arterial and capillary blood gas analysis, Bland–Altman’s plot, Regression model

## Abstract

One of the most feared neurological complications of premature birth is intraventricular hemorrhage, frequently triggered by fluctuations in cerebral blood flow (CBF). Although several techniques for CBF measurement have been developed, they are not part of clinical routine in neonatal intensive care. A promising tool for monitoring of CBF is its numerical assessment using standard clinical parameters such as mean arterial pressure, carbon dioxide partial pressure (pCO_2_) and oxygen partial pressure (pO_2_). A standard blood gas analysis is performed on arterial blood. In neonates, capillary blood is widely used for analysis of blood gas parameters. The purpose of this study was the assessment of differences between arterial and capillary analysis of blood gases and adjustment of the mathematical model for CBF calculation to capillary values. The statistical analysis of pCO_2_ and pO_2_ values collected from 254 preterm infants with a gestational age of 23–30 weeks revealed no significant differences between arterial and capillary pCO_2_ and significantly lower values for capillary pO_2_. The estimated mean differences between arterial and capillary pO_2_ of 15.15 mmHg (2.02 kPa) resulted in a significantly higher CBF calculated for capillary pO_2_ compared to CBF calculated for arterial pO_2_. Two methods for correction of capillary pO_2_ were proposed and compared, one based on the mean difference and another one based on a regression model.

*Conclusion*: Capillary blood gas analysis with correction for pO_2_ as proposed in the present work is an acceptable alternative to arterial sampling for the assessment of CBF.

**Table:** 

**What is Known:** *• Arterial blood analysis is the gold standard in clinical practice. However, capillary blood is widely used for estimating blood gas parameters.* *• There is no significant difference between the arterial and capillary pCO*_2_ *values, but the capillary pO*_2_ *differs significantly from the arterial one.*	
**What is New:** *• The lower capillary pO*_2_ *values yield significantly higher values of calculated CBF compared to CBF computed from arterial pO*_2_ *measurements.* *• Two correction methods for the adjustment of capillary pO*_2_ *to arterial pO*_2_ *that made the difference in the calculated CBF insignificant have been proposed.*

**What is Known:**

*• Arterial blood analysis is the gold standard in clinical practice. However, capillary blood is widely used for estimating blood gas parameters.*

*• There is no significant difference between the arterial and capillary pCO*_2_ *values, but the capillary pO*_2_ *differs significantly from the arterial one.*

**What is New:**

*• The lower capillary pO*_2_ *values yield significantly higher values of calculated CBF compared to CBF computed from arterial pO*_2_ *measurements.*

*• Two correction methods for the adjustment of capillary pO*_2_ *to arterial pO*_2_ *that made the difference in the calculated CBF insignificant have been proposed.*

## Introduction

One of the primary concerns in the care for preterm born infants is intraventricular hemorrhage (IVH), which may lead to death or permanent disabilities, such as cerebral palsy, learning disabilities, language disorders, blindness, and seizures. Preterm infants are at higher risk for IVH complications depending on their gestational age, weight, and additional risk factors usually occurring during the first 72 h of life until postnatal day seven. Infants born before 30 weeks of gestation (WG) or those with a birth weight below 1500 g are at risk with an overall incidence of 20 to 25% [1, 2]. In the preterm brain, IVH typically originates from the highly vascularized area of the germinal matrix [3, 4], which is present until 32 WG [5]. This crucial region for physiological fetal brain development contains numerous glial and neuronal precursor cells migrating to the cortex [3, 6]. Originating from the germinal matrix the hemorrhage may expand into the intraventricular space and/or into white and gray matter regions. Hemorrhages usually happen during the first 72 h of life until postnatal day seven.

Although risk factors of IVH are manifold, there is consensus that the most important ones are perinatal infection or inflammation, reduced coagulation profile, cardiovascular, circulatory and respiratory problems, electrolyte disturbances, mode and site of delivery, time of cord clamping, or genetic factors [6]. Deviations in mean arterial blood pressure (MAP), carbon dioxide pressure (pCO_2_), and cerebral blood flow (CBF) are significant clinical risk factors contributing to the rupture of germinal matrix vessels [7]. Blood gas disturbances in the early postnatal days of life are also suspected to induce brain injury and affect neurological outcome [8, 9]. Arterial pCO_2_ is one of the main regulators of CBF [10]. Both hypercarbia and hypocarbia are associated with complications like IVH, periventricular leukomalacia, and bronchopulmonary dysplasia [11]. Furthermore, hypoxia in combination with hypercarbia enforces a decrease in cerebrovascular resistance and a consecutive increase of CBF, which may lead to IVH [12].

Blood gases are usually analyzed in ventilated infants and those with non-invasive respiratory support. Further parameters like bilirubin, blood sugar, lactate, or electrolytes can be obtained from the same sample. There are several methods to get blood samples for gas analysis. Unfortunately, there are neither exact default values nor safe ranges for blood gas interpretation for preterm babies [13, 14]. Arterial blood gas measurements are the gold standard in clinical practice. Very preterm infants mostly receive catheters into the umbilical vessels (artery or vein); hence, arterial blood gas samples are available at least during the first days of life. Venous blood is drawn for, i.e., whole blood counts, coagulation, and liver enzymes. After the catheter is removed, capillary samples and transcutaneous measurements are widely used in order to reduce pain [15–18]. Transcutaneous devices are accurate in estimating gold standard arterial gases in neonatal ARDS (acute respiratory distress syndrome in term or near term born infants). However, due to skin problems (injuries from the transcutaneous device), they are not always available in very preterm infants. For the purpose of this study targeted at the most immature population of preterm infants, we only evaluated routinely taken capillary measurements.

Studies comparing arterial and capillary blood gas analysis in neonates have reported no significant differences for pCO_2_ in acceptable ranges [19–23]. Differences in pCO_2_ less than 1 kPa (0.04–1 kPa) are accepted and interpreted as clinically irrelevant [20, 23, 24]. For the other important parameter of our study, oxygen partial pressure (pO_2_), also good correlations have been shown; however, a systematic decrease of capillary pO_2_ values has been reported [22–25]. Studies measuring the difference between arterial and capillary values revealed clinical significance for a decrease of capillary pO_2_ in the range 1–3.3 kPa [24, 25]. Moreover, several studies have shown that the difference between arterial and capillary pO_2_ is even more significant for higher pO_2_ values [20, 21, 24].

The purpose of the present study was the statistical comparison of arterial and capillary measurements of pCO_2_ and pO_2_ obtained from 254 preterm infants and investigation of their effect on the numerical assessment of CBF. Furthermore, using regression analysis of clinical records two correction methods for the approximation of the arterial pO_2_ values by pO_2_ capillary values have been proposed, which allowed us to adjust the mathematical model for CBF calculation to capillary measurements. We demonstrated that following the adjustment, the difference between the CBF values calculated from capillary and arterial measurements was no longer statistically significant.

## Materials and methods

Data were retrospectively obtained from clinical records of 254 preterm infants treated in the Department of Neonatology at the University Hospital Essen and the Department of Pediatrics, School of Medicine, Klinikum rechts der Isar, Technical University of Munich. The study was approved by the ethical committee of the University Hospital Essen, University Duisburg-Essen (Ref. 16–7284-BO) and ethical committee of the School of Medicine, Klinikum rechts der Isar, Technical University of Munich (Ref. 364/15). The gestational age of the sample group ranged from 23 to 30 WG (26.45 ± 2.11 WG) and the body weight from 335 to 1580 g (864.06 ± 279.10 g). The occurrence of IVH was diagnosed by serial cranial ultrasound examinations. Patients without IVH (118) served as a control group and patients with IVH (136) as affected group. Basic clinical characteristics of the cohort are presented in Table 1, in which continuous variables are expressed as mean and standard deviation, while categorical variables are presented as the number of cases and percentages. MAP, pCO_2_, and pO_2_ were collected as routine clinical measurements during neonatal care for the first 10 days after birth in the control group, and for up to 7 consecutive days before and 3 days after hemorrhage (average of 6.7 ± 2.6 days) in the affected group. Dependent on the clinical situation either arterial or capillary measures were recorded. Capillary measurements were taken by arterialized (heel warmed) standard technique for preterm infants. In our study, arterial blood gas analysis is the gold standard, whereas capillary blood samples were compared to arterial ones. Since medical data were recorded during standard clinical care of the preterm infants, paired arterial and capillary blood measurements were not available. In order to improve the accuracy of statistical estimations and to reduce the influence of the patient’s clinical state, we paired consecutive arterial and capillary measurements carried out within several hours (in average 6.7 ± 4.6 h) during neonatal routine care. Measurements that could not be paired were excluded from analysis.

**Table 1:** Basic clinical parameters of the study population

Parameter		Whole cohort 254 (100%)	Control group 118 (100%)	Affected group 136 (100%)	*p*-value*
Gestational age (WG)		26.5 ± 2.1	26.7 ± 2.2	26.3 ± 2.0	0.1
Birth weight (g)		864.1 ± 279.1	850.7 ± 252.8	875.7 ± 300.5	0.7
Male		122 (48%)	48 (40.7%)	74 (54.4%)	0.03
Multiple birth		95 (37.4%)	43 (36.4%)	52 (38.2%)	0.8
APGAR	1 min	5.7 ± 2.2	6.2 ± 2.0	5.2 ± 2.2	< 0.01
	5 min	7.1 ± 1.7	7.5 ± 1.4	6.8 ± 1.8	< 0.01
	10 min	8.0 ± 1.2	8.4 ± 1.0	7.7 ± 1.4	< 0.01
Intubation during first 10 days [days]		4.7 ± 4.0	3.8 ± 4.1	5.5 ± 3.8	< 0.01
Neonatal bowel perforation (spontaneous/focal intestinal perforation SIP/FIP)		23 (9.1%)	3 (2.5%)	20 (14.7%)	< 0.01
Metabolic acidosis		23 (9.1%)	0	23 (16.9%)	< 0.01
EPH gestosis/preeclampsia		25 (9.8%)	18 (15.3%)	7 (5.1%)	0.01
Cholestasis		12 (4.7%)	1 (0.8%)	11 (8.1%)	0.01
Pulmonary hemorrhage		21 (8.3%)	5 (4.2%)	16 (11.8%)	0.04
Erythrocyte blood transfusion		164 (64.6%)	71 (60.1%)	93 (68.4%)	0.2
Sepsis		120 (47.2%)	50 (42.4%)	70 (51.5%)	0.2
Preterm premature rupture of membranes (PPROM)		73 (28.7%)	39 (33.1%)	34 (25.0%)	0.2
Chorioamnionitis/amniotic infection syndrome		117 (46.1%)	50 (42.4%)	67 (49.3%)	0.3
Pulmonary stenosis		4 (1.6%)	3 (2.5%)	1 (0.7%)	0.3
Intrauterine growth retardation (IUGR)		14 (5.5%)	8 (6.8%)	6 (4.4%)	0.4
Respiratory distress syndrome (RDS)		84 (33.1%)	42 (35.6%)	42 (30.9%)	0.5
In vitro fertilization (IVF)		32 (12.6%)	17 (14.4%)	15 (11.0%)	0.5
Disseminated intravascular coagulation (DIC)		2 (0.8%)	0	2 (1.5%)	0.5
Respiratory acidosis		1 (0.4%)	1 (0.8%)	0	0.5
Necrotizing enterocolitis (NEC)		20 (7.9%)	8 (6.8%)	12 (8.8%)	0.6
Feto-fetal transfusion syndrome (FFTS)		8 (3.1%)	4 (3.4%)	4 (2.9%)	1

^*^p-values are calculated for difference between control and affected groups

Mathematical evaluation of CBF was done by the hierarchical cerebrovascular model [26] with 19 levels describing different types of vessels (arteries, arterioles, capillaries, venules, and veins). The number and size of vessels on each level were adjusted to gestational age and birth weight of each individual infant [27]. The presence of the germinal matrix was simulated according to the gestational age [28] by the additional parallel compartment on the capillary level [27] with vessel’s density and size taken from the literature [29, 30]. CBF was calculated using Kirchhoff’s law as a ratio between cerebral perfusion pressure and total cerebral vascular resistance [31]. The latter was determined from the individual resistances of each vessel with accounting for diameter changes due to the vasoconstriction and vasodilation as a reaction on fluctuations of MAP [31], as well as changes in pCO_2_ [26] and pO_2_ [32] estimated from arterial blood samples. Cerebral perfusion pressure was estimated by the difference between clinically measured MAP and intracranial pressure. Since measurement of intracranial pressure is impossible, a constant value of 5 mmHg [33] was used for numerical calculations in all infants.

Statistical comparison of unpaired clinical parameters and calculated CBF was done using the two-sided Wilcoxon’s rank-sum test for continuous variables and Fisher’s exact test for categorical parameters. In both cases, 5% significance level was set. For statistical comparison of paired measurements, a paired-sample *t*-test with the same significance level was used. For adjustment of capillary blood measurements, linear regression analysis and Bland–Altman’s plot [34–36] were applied. Both methods are widely used in clinical studies [37, 38] to assess the agreement and accuracy between two techniques. The basic concept of Bland–Altman’s approach is the visualization of the difference of the measurements made by two methods. When differences are symmetrical around zero, there are no systematic bias. The most common way to construct Bland–Altman’s plot, when neither of methods is “reference,” is plotting the difference between the two paired measurements against their mean value. However, it is also possible to use either of the measurements, reference or actual, instead of their mean value [39]. The present study aimed to approximate arterial values using available capillary values; therefore, the difference between arterial and capillary blood measurements was plotted against the available capillary value. In addition, to estimate the proportional bias, the Bland–Altman’s analysis was combined with a linear regression analysis [36, 40]. To assess the significance of the linear regression model, we used *F*-statistic versus constant model with a significance level of 5%. All statistical methods were taken from the standard MATLAB2020a library.

## Results

**Fig. 1:**
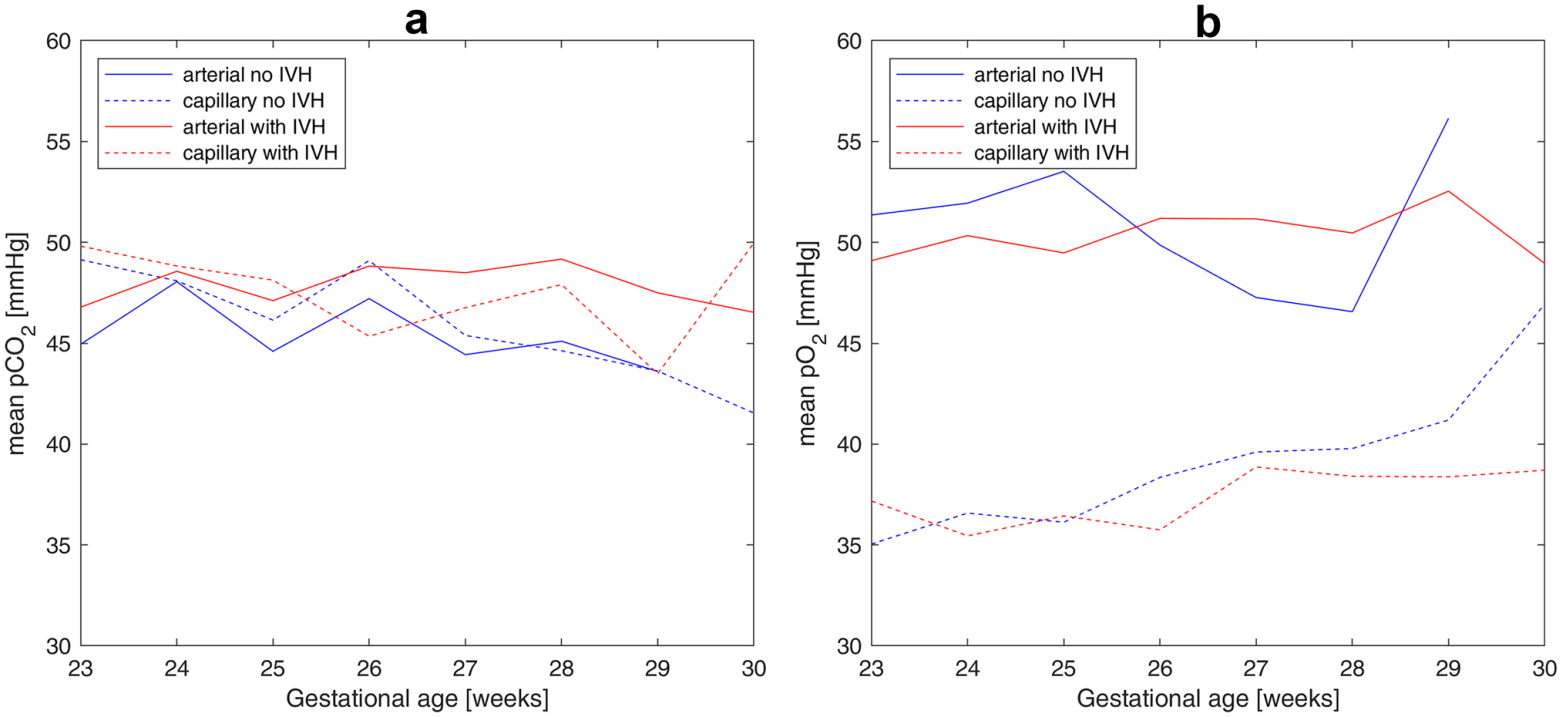
Mean values of the arterial and capillary blood measurements of pCO_2_ (**a**) and pO_2_ (**b**) versus gestational age

**Fig. 2:**
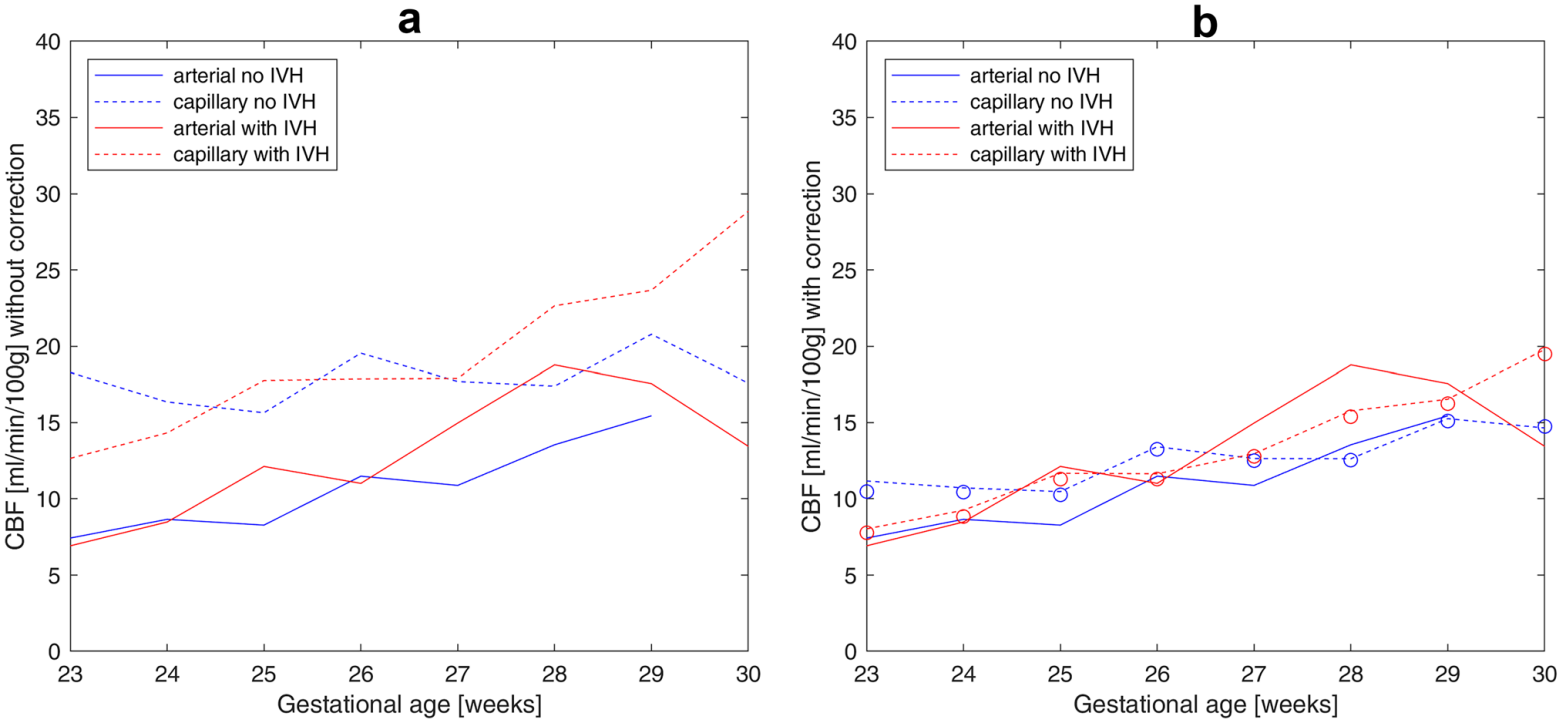
Mean values of CBF calculated from arterial (solid lines) and capillary (dashed lines) blood measurements versus gestational age: **a** capillary pO_2_ values without correction; **b** capillary pO_2_ measurements corrected with mean difference (dashed lines without symbols: pO_2_^cap^ + 15.15 mmHg) or with linear regression (circles: 0.64·pO_2_^cap^ + 28.32 mmHg)

In the analyzed data set, the number of arterial and capillary gas measurements depended on gestational age and IVH diagnosis (Table 2). For gestational ages of 23–24 weeks, there were more arterial than capillary blood measurements available, in both the control and the affected group. Starting from 25 WG, capillary measurements in the control group dominated over arterial ones, while a dominance of the capillary measurements in the affected group was observed for 30 WG only. Statistical analysis of unpaired arterial and capillary blood measurements revealed that mean values (Fig. 1) significantly differed for pO_2_, but not for pCO_2_ (Table 3). As a consequence, values of CBF calculated with our model from capillary blood measurements (Fig. 2a) were significantly higher than those calculated from arterial analyses for both control and affected groups (Table 5).

**Table 2:** Number of arterial and capillary blood measurements

Weeks of gestation	No IVH				With IVH		
	Arterial	Capillary	Paired	Arterial		Capillary	Paired
23	164	114	26	293		90	26
24	327	194	26	315		154	51
25	139	227	26	272		192	47
26	104	250	14	291		135	19
27	89	150	11	119		112	12
28	11	223	0	162		123	18
29	8	104	0	105		87	14
30	0	102	0	6		72	3
All	842	1364	103	1563		965	190

**Table 3:** Mean values and standard deviations of arterial and capillary blood gases

Parameter	Unpaired measurements				Paired measurements				
	Arterial	Capillary	*p*-value*	Mean (art-cap)	Arterial		Capillary	*p*-value**	Mean (art-cap)
pCO_2_ (mmHg)	47.41 ± 10.93	46.84 ± 9.53	0.11	0.57	45.90 ± 10.16		46.42 ± 9.13	0.38	−0.52
pO_2_ (mmHg)	50.67 ± 12.75	38.04 ± 7.78	< 0.001	12.63	52.11 ± 11.84		36.96 ± 5.85	< 0.001	15.15

^*^p-values are calculated using two-sided Wilcoxon’s rank-sum test

^**^p-values are calculated using paired-sample *t*-test

**Fig. 3:**
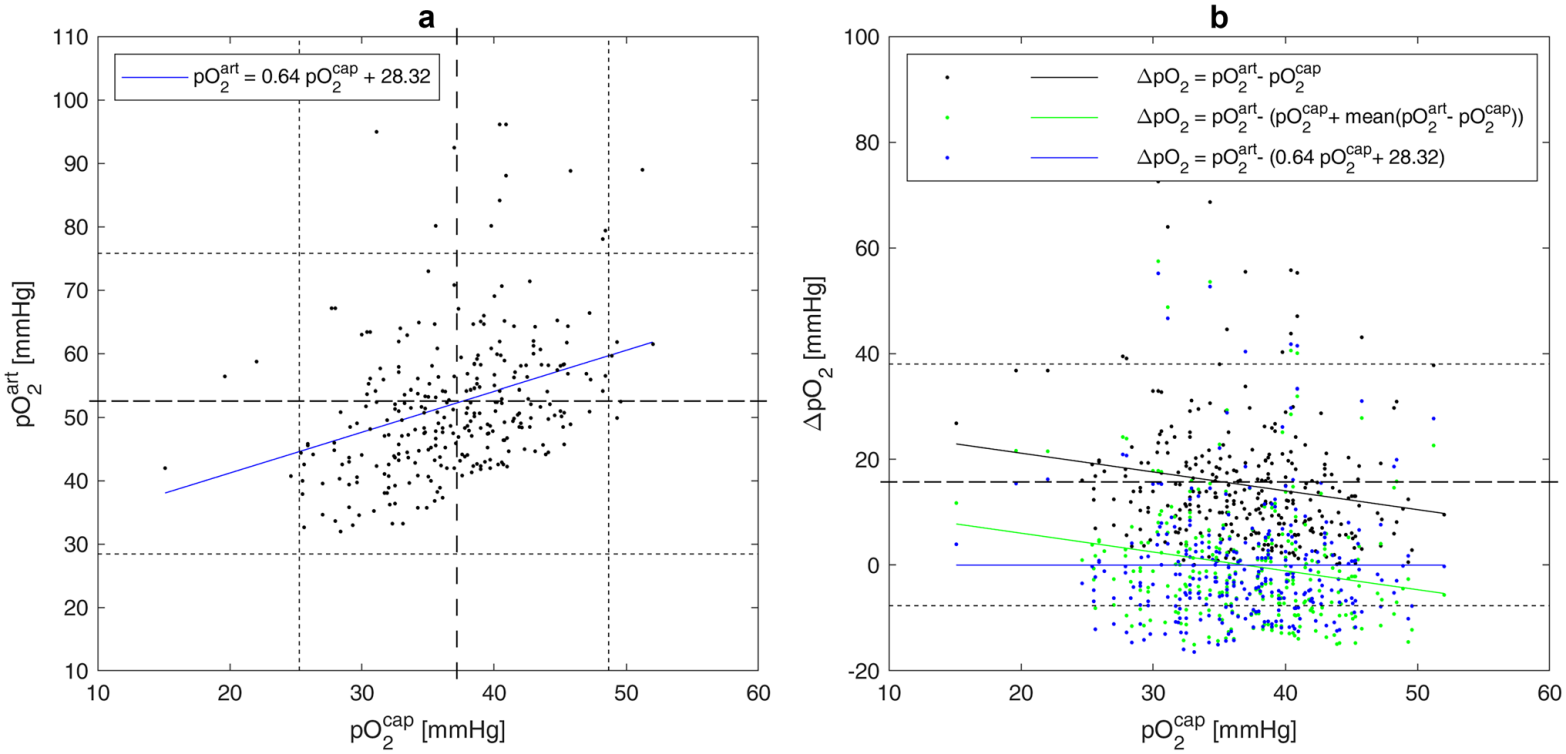
**a** Scatter plot for capillary and arterial measurements of pO_2_. Dashed and dotted lines indicate mean and mean ± 2 standard deviation of variables on the corresponding axis. **b** Bland–Altman plot for the difference between capillary and arterial measurements of pO_2_. Dashed and dotted lines indicate mean difference and 95% limits of agreement for the difference pO_2_^art^ − pO_2_^cap^

In order to assess the quantitative difference between arterial and capillary pO_2_ values, we analyzed 293 paired consecutive records collected from both groups (Table 2). The standard scatter plot for capillary (pO_2_^cap^) and arterially (pO_2_^art^) measurements and corresponding linear regression line are shown in Fig. 3a. Confidence intervals (dotted lines) were calculated as mean ± 2 standard deviation of pO_2_^cap^ or pO_2_^art^, respectively. Regression line (solid blue line) has a slope of 0.64 and an intercept of 28.32 mmHg (*R*^2^ = 0.1, *p* < 0.001). The corresponding Bland–Altman plot (Fig. 3b) was constructed as a scatter plot for pO_2_^cap^ and difference pO_2_^art^ − pO_2_^cap^ (black dots). The Bland–Altman plot (Fig. 3b) demonstrated significant differences between capillary and arterial pO_2_ measurements, which resulted in a systematic bias of 15.15 mmHg (dashed line) (Table 3). As a measure of precision, the 95% limits of agreement are shown (dotted lines in Fig. 3b). Most of the points are located within the limits of agreement (calculated as mean ± 2 standard deviation of the difference pO_2_^art^ − pO_2_^cap^). All 13 outliers in Fig. 3b are located over the upper limit of agreement and correspond to the outliers of arterial measurements in Fig. 3a, which lie over the upper limit of the confidence interval of pO_2_^art^.

Plotting the differences pO_2_^art^ − pO_2_^cap^ against the capillary measurement pO_2_^cap^ and performing linear regression analysis, we obtained the regression line (black solid line in the Fig. 3b) with statistically significant nonzero intercept and slope (Table 4) indicating the presence of both systematic and proportional bias reflecting statistically significant trend in the data. Systematic bias (Table 4) means that pO_2_^cap^ values are consistently lower than pO_2_^art^ values across the whole range of measurement with the mean difference of 15.15 mmHg. Negatively proportional bias indicates that the difference between two measuring methods increases with decreasing pO_2_^cap^.

**Table 4:** The difference between arterial and capillary measurements of pO_2_ using linear regression models

Difference (mmHg)	Mean (mmHg)	Intercept (mmHg)	Slope	*p*-value*
pO_2_^art^ − pO_2_^cap^	15.15	28.32	−0.36	0.002
pO_2_^art^ − pO_2_^pM^	5.2·10¯^15^	13.17	−0.36	0.002
pO_2_^art^ − pO_2_^pR^	−4·10¯^16^	−1·10¯^14^	2·10¯^16^	1

^*^p-values are calculated using *F*-statistic versus constant model

The first approach to adjust capillary data is to add the mean value of difference pO_2_^art^ − pO_2_^cap^ to the measured value: pO_2_^pM^ = pO_2_^cap^ + 15.15 mmHg. The resulting corrected values pO_2_^pM^ have the difference pO_2_^art^ − pO_2_^pM^ with arterial measurements (green dots in Fig. 3b) with the mean value of 5.2·10¯^15^. However, the regression line of the difference pO_2_^art^ − pO_2_^pM^ (solid green line in Fig. 3b) still has a nonzero slope and intercept (Table 4), meaning that proportional bias is still present. A better adjustment of capillary measurements can be obtained using linear regression between pO_2_^cap^ and pO_2_^art^ (Fig. 3a) as follows: pO_2_^pR^ = 0.64·pO_2_^cap^ + 28.32 mmHg. As a result, the mean difference between pO_2_^art^ and pO_2_^pR^ decreases to zero (Table 4) and mean value of pO_2_ increases from 36.96 ± 5.85 mmHg up to 51.97 ± 3.76 mmHg. The estimated mean difference and linear regression coefficients can be further used for the correction of the bias between arterial and capillary measurements in the numerical calculation of CBF.

The CBF values calculated for arterial, capillary, and corrected capillary values are compared in Table 5. Since our statistical analysis revealed no significant differences between arterial and capillary blood measurements for pCO_2_ (Table 3), the estimated for pCO_2_ mean difference of −0.51 mmHg (−0.07 kPa) was neglected when calculating CBF. Correction of capillary pO_2_ was carried out using three methods: (1) with mean value of unpaired measurements, pO_2_^upM^ = pO_2_^cap^ + 12.63 mmHg (correction I); (2) with mean value of paired measurements: pO_2_^pM^ = pO_2_^cap^ + 15.15 mmHg (correction II); and (3) with linear regression coefficients: pO_2_^pR^ = 0.64·pO_2_^cap^ + 28.32 mmHg (correction III). After correction, the difference between CBF calculated from arterial and capillary measurements (Fig. 2b) was no longer significant both in control and affected groups (*p*_*a*_-values in Table 5). Furthermore, the difference between correction methods was also not significant in both groups (*p*_dif_-values in Table 5). Although the difference between mean values of CBF in control and affected groups was higher for arterial measurements than for capillary ones (both uncorrected and corrected), it was insignificant for all kinds of pO_2_ values (*p*_IVH_-values in Table 5).

**Table 5:** Mean values and standard deviations of CBF calculated from arterial and capillary blood measurements without and with correction of capillary pO_2_. The *p*_*a*_-values* are calculated versus CBF computed using pO_2_^art^. The *p*_IVH_-values* are calculated for the difference between control and affected groups. The *p*_dif_-values* are calculated for the difference in correction methods

pO_2_ (mmHg)	CBF (ml/100 g/min) No IVH	CBF (ml/100 g/min) With IVH	*p*_IVH_
pO_2_^art^	8.99 ± 4.61	11.42 ± 9.33	0.39
pO_2_^cap^ (no correction)	17.76 ± 8.76 *p*_*a*_ < 0.001	18.76 ± 11.18 *p*_*a*_ = 0.02	0.51
pO_2_^upM^ (correction I)	12.82 ± 5.57 *p*_*a*_ = 0.15	13.13 ± 7.19 *p*_*a*_ = 0.79	0.79
pO_2_^pM^ (correction II)	12.43 ± 5.29 *p*_*a*_ = 0.34 *p*_dif_ = 0.58 (corrections II and I)	12.65 ± 6.79 *p*_*a*_ = 0.96 *p*_dif_ = 0.65 (corrections II and I)	0.79
pO_2_^pR^ (correction III)	12.24 ± 5.06 *p*_*a*_ = 0.39 *p*_dif_ = 0.44 (corrections III and I) *p*_dif_ = 0.57 (corrections III and II)	12.32 ± 6.45 p_a_ = 0.96 *p*_dif_ = 0.57 (corrections III and I) *p*_dif_ = 0.65 (corrections III and II)	0.79

^*^p_*a*_, *p*_IVH_, and *p*_dif_ values are calculated using two-sided Wilcoxon’s rank-sum test

## Discussion

The main goal in the treatment of preterm infants is to avoid cerebral hemorrhage and its neurological consequences. CBF plays a significant role in the development of IVH, but is still not routinely measured during neonatal clinical care of preterm infants. Therefore, the aim of the present study was to adjust a mathematical model for CBF calculation for data obtained by regular analysis from arterial and capillary blood gas samples. Linear regression analysis and Bland–Altman’s plot were used to assess the correspondence between the two methods of blood sampling. Statistical analyses of clinical records of 254 preterm infants revealed no significant differences between the values of pCO_2_ obtained from arterial or capillary blood samples, what is in agreement with previous findings [20, 22, 25] that capillary blood measurements of pCO_2_ correctly predict arterial values. Likewise, Tan [41] has confirmed that except for pO_2_ venous and capillary blood gases in neonates are well correlated and mostly interchangeable with arterial values.

In the present data set, capillary pO_2_ values were significantly lower than arterial ones and CBF values calculated from the capillary pO_2_ were significantly higher than that computed from arterial pO_2_. This is in agreement with previous observations [42, 43] that due to the cerebral autoregulation, a decrease in pO_2_ leads to vasodilation and, as a consequence, to a linear increase in CBF. To adjust capillary measurements to arterial ones, statistical analysis was performed in two ways: for unpaired and paired measurements. In both cases, capillary blood analyses of pO_2_ showed systematic and proportional bias in comparison to arterial ones. It is important to underline that the mean difference between arterial and capillary pO_2_ was similar in both approaches, namely, 12.63 for unpaired data and 15.15 for paired data. The estimated mean difference of 15.15 mmHg (2.03 kPa) was close to published discrepancies of 2.17 kPa (16.28 mmHg) and 2.47 kPa (18.53 mmHg) evaluated from 158 paired arterial and capillary blood samples being obtained from warmed and not warmed heels, respectively [24]. However, these values are lower than the difference of 30.2 mmHg (4.03 kPa) estimated from capillary blood samples drawn from 21 preterm infants using the Tenderfoot automated capillary sampling device [22]. The estimated mean difference between capillary and arterial pO_2_ values was added to the capillary pO_2_ measurement. Two corrections, pO_2_^upM^ obtained from unpaired measurements and pO_2_^pM^ obtained from paired measurements, have reduced systematic bias, but not the proportional one. A better adjustment was obtained using correction with linear regression coefficients. We assumed that paired measurements are more reliable than unpaired ones and for this reason used paired measurements for regression analyses. The correction pO_2_^pR^ compensated both systematic and proportional bias for differences between capillary and arterial pO_2_.

Three correction methods were used in the mathematical calculation of CBF, and the difference between results was insignificant. Mean CBF values calculated using pO_2_^upM^, pO_2_^pM^, and pO_2_^pR^ were decreased in comparison to that computed using uncorrected capillary pO_2_ values. The difference between CBF calculated using arterial and corrected capillary pO_2_ was proven to be statistically insignificant. Thus, the artificial increase of calculated CBF due to the uncorrected capillary pO_2_ has been compensated by the proposed statistical correction methods. The comparison of CBF between control and affected groups revealed that CBF was insignificantly higher in the affected group both for arterial and capillary (uncorrected and corrected) pO_2_.

Although the regression model was superior in reduction of bias, all correction methods have resulted in statistically equal CBF values calculated from arterial and corrected capillary measurements, with no significant difference between correction methods. Thus, the correction pO_2_^pR^ can be proposed for mathematical calculation of CBF, while the correction pO_2_^pM^ can be suggested as a simple and convenient approximation of arterial measurement for practical application.

## Limitations of the study

The most important limitations of the present study arose from its retrospective nature. Medical records were evaluated in two different hospitals over a period of 10 years. During this time, the blood sampling technique and arterialization process may have changed, although its effect on capillary measurements is still under debate [24, 25]. In order to minimize the effects of data collection methods, arterial and capillary measurements from the same patient were analyzed. The main disadvantage was the lack of paired blood gas measurements. Pairs of arterial and capillary measurements were formed from the successive records of clinical routine data. As a consequence, time intervals between measurements were up to several hours, which could increase estimated errors. However, statistical values obtained from 4734 unpaired measurements were close to those of paired measurements. Besides, 293 pairs of measurements from 254 infants analyzed in the current study clearly outnumbered data sets from pervious clinical studies in 21 [22] or 41 [24] preterm infants.

Another limitation of the study was the lack of a comparison between calculated and measured values of CBF. Although near infrared spectroscopy (NIRS) and Doppler ultrasound are available in modern neonatal intensive care units to evaluate cerebral perfusion, their use is dependent on special clinical situations. Doppler ultrasound is used in critically ill infants (i.e., following severe intracerebral hemorrhage or for exclusion of cerebral edema). NIRS is used mainly for research purposes and for special clinical situations such as asphyxia in term born infants.

The main purpose of the present study was the adjustment of the mathematical model for CBF calculation to capillary measurements. Since other clinical parameters such as oxygen saturation SO_2_, pH, hematocrit, thrombocyte count, and indicators of inflammation are not yet included in this model, they were not analyzed. It is planned to account for them in further extensions of the mathematical model.

## Conclusion

The present study has compared arterial and capillary blood gas analysis, investigated the effect of capillary measurements of pCO_2_ and pO_2_ on calculated CBF, and adjusted the mathematical model for CBF calculation to capillary blood analyses. Following the adjustment, the difference between CBF values calculated from capillary and arterial samples became statistically insignificant. Therefore, we state that capillary blood analyses with correction for pO_2_ as proposed in the present work is an acceptable alternative to arterial sampling for assessment of CBF. Thus, this mathematical model may help to predict critical CBF values allowing early identification of preterm infants at risk for IVH.

## Data Availability

The dataset supporting the conclusions of this article is available at the mediaTUM, publications repository of the Technical University of Munich, https://mediatum.ub.tum.de/1521896.

## Code availability

All calculations presented in the paper were produced with the standard software package MATLAB2020a.

## Abbreviations

CBF: Cerebral blood flow
IVH: Intraventricular cerebral hemorrhage
MAP: Mean arterial pressure
pCO_2_: Carbon dioxide partial pressure
pO_2_: Oxygen partial pressure
WG: Week of gestation
